# Innovative Regression Model for Frequency-Dependent Acoustic Source Strength in the Aquatic Environment: Bridging Scientific Insight and Practical Applications

**DOI:** 10.3390/s25051560

**Published:** 2025-03-03

**Authors:** Moshe Greenberg, Uri Kushnir, Vladimir Frid

**Affiliations:** Civil Engineering Department, Sami Shamoon College of Engineering, Jabotinsky 84, Ashdod 7747629, Israel; moshegr1@ac.sce.ac.il (M.G.); uriku@ac.sce.ac.il (U.K.)

**Keywords:** sound attenuation, nonlinear regression, acoustic source strength, marine environment

## Abstract

This study addresses the challenge of predicting acoustic source strength in freshwater environments, focusing on frequencies between 100–400 kHz. Acoustic signal attenuation is inherently frequency-dependent and influenced by water properties as well as the total propagation path of the acoustic wave, complicating the accurate determination of source strength. To address this challenge, we developed a non-linear regression model for solving the inverse problem of attenuation correction in reflected signals from typical aquatic reflectors, addressing the current absence of robust correction tools in this frequency range. The novelty of our approach lies in designing a non-linear regression framework that incorporates key physical parameters—signal energy, propagation distance, and frequency—enabling accurate source strength prediction. Using an experimental setup comprising ultrasonic transducers and a signal generator under controlled conditions, we collected a comprehensive dataset of 366 samples. The results demonstrate that our proposed model achieves reliable source strength prediction by simplifying Thorpe’s equation for freshwater environments. This research represents a significant advancement in underwater acoustics, providing a practical and reliable tool for source strength estimation in freshwater systems. The developed methodology may have broad applications across sonar technology, environmental monitoring, and aquatic research domains.

## 1. Introduction

Acoustic reflectors in the marine environment, such as seabed sediments and artificial targets, exhibit unique reflection spectra [1,2]. These spectral characteristics arise from the interaction between the acoustic wavelength and the physical attributes of the reflector, including grain size, voids, inclusions, and layering. This interaction enables the classification of seabed materials based on their acoustic response. However, a significant challenge arises due to the frequency-dependent attenuation of acoustic signals, which affects both the transmitted and reflected components of the signal. The reflection spectrum is the ratio of the incident amplitude (or energy) to the reflected amplitude (or energy) at each frequency. However, since attenuation is frequency-dependent, the signal recorded by the receiver is not an exact representation of the reflected signal, just as the transmitted signal is not identical to the incident signal. In effect, water acts as a frequency-dependent filter, altering the spectral composition of the signal during propagation. While the attenuation of the incident signal can be pre-measured at controlled distance intervals, as it is independent of the reflector, this approach does not apply to the reflected signal. Therefore, a dedicated method is required to correct attenuation effects by solving the inverse signal reconstruction problem to enable reliable classification based on reflection spectra.

The frequency-dependent attenuation of sound in water is a fundamental concept in underwater acoustics and is essential for applications such as sonar, communication, and environmental monitoring. Foundational works [1,2] demonstrated that attenuation is influenced by chemical relaxation processes, particularly involving boric acid and magnesium sulfate, which vary significantly with frequency. This chemical and molecular interaction, especially at higher frequencies, introduces nonlinear attenuation effects, a relationship further quantified by Fisher and Simmons [3], who measured sound absorption over a range of frequencies. Later, Zhou & Luo [4] expanded on these foundational studies through finite-element modeling, analyzing how sedimentary layers influence sound attenuation in underwater environments. Their work highlights the role of high-frequency scattering and reflections from seabed sediments, creating complex acoustic propagation patterns. Hamilton [5] and Kinsler et al. [6] provided additional insights into how seabed and water column interactions alter attenuation in shallow waters, with prominent multi-path effects. This work has laid the groundwork for understanding sound attenuation in environments with variable sediment compositions and supports predictions used in practical applications across diverse marine conditions.

Van Komen et al. [7] introduced a notable advancement in computational techniques, which used convolutional neural networks (CNNs) trained on large datasets to predict frequency-dependent attenuation across diverse marine environments. Their approach effectively captured nuanced environmental impacts that traditional models may overlook. Expanding on these methods, Ruan and Zhou [8] applied deep-learning techniques to address the complexity of seabed topographies and reported significantly improved accuracy in high-frequency attenuation predictions. Jakacki et al. [9] further demonstrated how data assimilation techniques enable real-time model adaptation, which is crucial for dynamic marine environments with rapidly changing conditions. Their work on high-resolution ocean modeling highlights the potential of computational methods in refining frequency-dependent attenuation models, paving the way for more accurate underwater acoustic predictions. Environmental and biological factors further contribute to frequency-dependent attenuation. Richards & Leighton [10] conducted high-resolution measurements in coastal waters, showing that microbubbles significantly amplify sound absorption, particularly in high-frequency ranges and turbulent areas. Liu & Li [11] provided insight into microbubble resonance phenomena, emphasizing how these interactions lead to higher sound attenuation in biologically active, turbulent waters. Kahn [12] extended these findings to brackish and freshwater systems, revealing that high concentrations of suspended particles, including silt and organic debris, also increase attenuation at higher frequencies. These studies underscore the complexity of frequency-dependent attenuation and the need for environment-specific models that account for biological and particulate factors in marine acoustics. Beyond theoretical modeling, underwater acoustic research has played a crucial role in marine conservation and policy. Guan et al. [13] emphasized the importance of accurate seabed characterization in regulatory impact assessments, highlighting how sound propagation models must account for substrate layering and acoustic impedance to ensure reliable environmental predictions.

Advanced molecular absorption models have also been refined. Richards [14] updated the boric acid relaxation model initially proposed by Francois and Garrison [2], addressing dynamic chemical responses in variable salinity and temperature conditions and refining its application in turbid seawater environments. Such refinements improve attenuation predictions in coastal regions, where environmental variability often complicates acoustic signal transmission. These advanced models, particularly those that integrate real-time environmental data, provide high accuracy in predicting sound absorption across frequency bands, which is essential for underwater communication and navigation systems.

Solving the inverse problem of determining acoustic source strength from an attenuated signal is critical for underwater navigation, sonar, and monitoring applications. Early foundational work by Munk and Wunsch [15] and Tarantola [16] developed theoretical frameworks for reconstructing sound fields in complex marine environments, addressing the challenge of multi-path reflections and environmental noise. Dettmer et al. [17] expanded on this by applying Bayesian inversion frameworks to improve source strength estimates, particularly in noisy and dynamically changing environments. Their trans-dimensional inversion approach allows adaptive parameter estimation, enhancing acoustic modeling accuracy. Gunes & Guldogan [18] integrated Monte Carlo simulations with frequency-domain modeling, enabling robust source strength estimation by accounting for environmental variability.

Regularization methods have been critical in enhancing inversion accuracy under frequency-dependent attenuation. Kirkeby et al. [19] introduced adaptive regularization techniques that adjust based on frequency, optimizing inversion accuracy in shallow water environments, which had strong attenuation effects and multi-path reflections that complicated source estimation. Hansen [20] applied Tikhonov regularization, a widely used method in acoustic inversion, to mitigate noise interference, making it suitable for various ocean acoustic applications.

Machine learning has recently been applied to the inverse problem. Chen and Schmidt [21] used neural networks trained on synthetic datasets to accurately estimate source strength, even in environments with complex attenuation profiles. This approach reduces the computational demands of traditional iterative inversion methods and shows promise for real-time applications in acoustically complex underwater environments. Dostoevsky and Rogers [22] extended these methods with a deep reinforcement learning framework that dynamically adjusts inversion parameters to maximize reconstruction fidelity, making real-time solutions feasible in dynamic marine environments. Recent acoustic signal processing advancements have refined approaches for nonlinear underwater acoustic signals. Campo-Valera et al. [23] demonstrated the effectiveness of parametric signal processing for enhancing Time of Arrival (ToA) estimation and amplitude correction in underwater communications. These advancements provide a strong foundation for addressing frequency-dependent attenuation in seabed classification, particularly in the presence of nonlinear signal distortions.

Numerical simulation advancements have also improved the accuracy of inverse problem solutions. Adams et al. [24] employed high-resolution finite-difference time-domain (FDTD) simulations to model multi-path reflections and frequency-dependent scattering, enhancing inversion models by integrating environment-specific parameters. Manduzio et al. [25] demonstrated the efficacy of these methods in coastal environments, where adaptive inversion techniques address variable sound speed profiles and multi-path reflections, significantly improving source strength estimations.

Recent studies have integrated data assimilation with inverse problem-solving to adapt to real-time environmental changes. Storto et al. [26] showed that continuously updating sound attenuation parameters with real-time environmental data enhances inversion accuracy, especially in dynamic marine conditions. This approach is highly effective for sonar and tracking applications, where precise source strength estimation is essential for reliable navigation and detection. By merging classical inversion techniques with data-driven and environment-specific models, current methods have achieved robust solutions for determining acoustic source strength across frequency ranges in complex underwater settings.

Many aspects of sound attenuation in water and contributing effects can also be found in [27].

The present study aims to develop a regression-based model that accurately predicts acoustic source strength within the 100–400 kHz frequency range in freshwater environments. This model will address the inverse problem of attenuation correction for reflected signals by integrating key physical parameters—signal energy, propagation path length, and frequency. By leveraging a dataset of 366 controlled measurements, we propose a non-linear regression framework capable of providing reliable source strength estimations, offering a novel and practical alternative to traditional attenuation correction methods.

The paper is organized as follows: Section 2 describes the Regression Models and the methodology used for data collection and regression modeling. Section 3 describes the experimental setup and the data set for this research. Section 4 presents the results of the proposed model, including accuracy assessment and comparisons with existing approaches. Section 5 discusses the findings’ implications, limitations, and potential future research directions. Finally, Section 6 provides concluding remarks and highlights the broader significance of this study in underwater acoustics.

## 2. Regression Models

The regression model predicts the result value Yp (source energy in our case) as a nonlinear combination of the features comprising the entries of the design matrix X. The training process searches for the nonlinear combination of features that minimize the mean square error defined by

(1) $$MSE=\sum_{1}^{Mt}{\left(Y_{p}-Y\right)}^{2}$$

where Y is the measured result, Yp is the predicted result, and $\sum_{1}^{Mt}$ indicates summation of the training set. Nonlinearities can be introduced using feature engineering. The features also indicate the assumed physical model of sound attenuation (or its inverse).

### Feature Choice for the Model—Physical Attenuation Models

The strength loss of the signal (transmission loss, TL) is defined as follows [28]:

(2) $$TL=-10log(\frac{I_{i}}{I_{0}})$$

where $I_{i}$ is the signal intensity (Energy per unit surface) at a distance $R_{i}$ and $I_{0}$ is the signal intensity at a reference distance $R_{0}$. The negative sign indicates a reduction in acoustic intensity with propagation.

The transmission loss is comprised of two main components, namely, geometric spreading and absorption.

Assuming the wavefront area (either spherical or other) is proportional to the distance squared and conservation of energy, we obtain the following:

(3) $$I_{0}R_{0}^{2}=I_{i}R_{i}^{2}$$

Hence, the geometric part of the transmission loss is given by:

(4) $$GTL=-20log(\frac{R_{i}}{R_{0}})$$

Another mechanism by which the acoustic energy decays with propagation is absorption due to shear viscosity, volume viscosity, and ion relaxation (seawater). The absorption transmission loss is given by:

(5) ATL=−αR

where α is the absorption coefficient, which is frequency dependent. A general equation for α is given by [25]:

(6) $$\alpha =\Sigma A_{i}\frac{f^{2}a_{i}}{f^{2}+a_{i}^{2}}+bf^{2}$$

where f, is the frequency and Ai and $a_{i}$, are constant. The widely used equation by Thorpe [28] is a private case of this formula. Commonly, there are three arguments in the summation in Equation (6) expressing the ion relaxation associated with (in seawater) magnesium sulfate, boric acid, and magnesium carbonate. Substituting the geometric loss and the absorption loss into the transmission loss equation, we get:

(7) $$-10\log\left(\frac{I_{i}}{I_{0}}\right)=20\log\left(\frac{R_{i}}{R_{0}}\right)+\left[A_{1}\frac{f^{2}a_{1}}{f^{2}+a_{1}^{2}}+A_{2}\frac{f^{2}a_{2}}{f^{2}+a_{2}^{2}}+A_{3}\frac{f^{2}a_{3}}{f^{2}+a_{3}^{2}}+bf^{2}\right]\left(R_{i}-R_{0}\right)$$

Assuming $R_{0}\;$= 1, reorganizing and generalizing unknown constants to be determined by the regression model we obtain:

Case 0

(8) $$\log\left(I_{0}\right)=C_{1}\log\left(I_{i}\right)+C_{2}\log\left(R_{i}\right)+\left[C_{3}f^{2}+C_{4}\frac{f^{2}c_{4}}{f^{2}+c_{4}^{2}}+C_{5}\frac{f^{2}c_{5}}{f^{2}+c_{5}^{2}}+C_{6}\frac{f^{2}c_{6}}{f^{2}+c_{6}^{2}}\right]\left(R_{i}-1\right)+C_{7}$$

where the result vector entries are measured $\log\left(I_{0}\right)$ values, the features X are $log\left(I_{i}\right),log\left(R_{i}\right),f^{2}\cdot R,\;f^{2},\;I_{i}\;,\;f\;,\;f\cdot R$ and the constants to be determined are ${C_{1},C_{2},C}_{3},C_{4},C_{5},C_{6},C_{7},c_{4},c_{5},c_{6}$.

Since Thorpe developed his equation for saltwater and large distances, additional terms can be introduced to accommodate for near-field behavior, such as presented for cases 1 to 4:

Case 1

(9) $$\log\left(I_{0}\right)=C_{1}\log\left(I_{i}\right)+C_{2}\log\left(R_{i}\right)+\left[C_{3}f^{2}+C_{4}\frac{f^{2}c_{4}}{f^{2}+c_{4}^{2}}+C_{5}\frac{f^{2}c_{5}}{f^{2}+c_{5}^{2}}+C_{6}\frac{f^{2}c_{6}}{f^{2}+c_{6}^{2}}\right]\left(R_{i}-1\right)+C_{7}+C_{8}I_{i}$$

Case 2

(10) $$\log\left(I_{0}\right)=C_{1}\log\left(I_{i}\right)+C_{2}\log\left(R_{i}\right)+\left[C_{3}f^{2}+C_{4}\frac{f^{2}c_{4}}{f^{2}+c_{4}^{2}}+C_{5}\frac{f^{2}c_{5}}{f^{2}+c_{5}^{2}}+C_{6}\frac{f^{2}c_{6}}{f^{2}+c_{6}^{2}}\right]\left(R_{i}-1\right)+C_{7}+C_{9}f$$

Case 3

(11) $$\log\left(I_{0}\right)=C_{1}\log\left(I_{i}\right)+C_{2}\log\left(R_{i}\right)+\left[C_{3}f^{2}+C_{4}\frac{f^{2}c_{4}}{f^{2}+c_{4}^{2}}+C_{5}\frac{f^{2}c_{5}}{f^{2}+c_{5}^{2}}+C_{6}\frac{f^{2}c_{6}}{f^{2}+c_{6}^{2}}\right]\left(R_{i}-1\right)+C_{7}+C_{8}I_{i}+C_{9}f$$

Case 4

(12) $$\log\left(I_{0}\right)=C_{1}\log\left(I_{i}\right)+C_{2}\log\left(R_{i}\right)+\left[C_{3}f^{2}+C_{4}\frac{f^{2}c_{4}}{f^{2}+c_{4}^{2}}+C_{5}\frac{f^{2}c_{5}}{f^{2}+c_{5}^{2}}+C_{6}\frac{f^{2}c_{6}}{f^{2}+c_{6}^{2}}\right]\left(R_{i}-1\right)+C_{7}+C_{8}I_{i}+C_{9}f+C_{10}f\left(R_{i}-1\right)$$

The distinctions between Case 0 and Case 4 arise from a progressive refinement of the regression model to better capture the physical phenomena governing acoustic attenuation in freshwater environments. Initially, Case 0 serves as a baseline, incorporating only fundamental logarithmic and quadratic terms that reflect basic geometric spreading and absorption effects. Recognizing that short-range propagation may deviate from ideal spherical spreading, subsequent cases introduce additional correction terms: for example, Case 1 incorporates adjustments for short signal travel distances behavior, while Case 2 adds terms to better capture the nonlinear, frequency-dependent absorption observed at high frequencies. Cases 3 and 4 refine the model by integrating extra empirical corrections that adjust for subtle discrepancies between theoretical predictions (such as those based on Thorpe’s equation) and experimental observations. The rationale behind these classifications was guided by iterative testing and evaluation using mean squared error (MSE) as a performance metric, ensuring that a corresponding improvement in model accuracy justified each additional term. This hierarchical approach allowed us to systematically address the limitations of simpler models and ultimately select the configuration that best represents the complex interplay between geometric spreading, absorption, and near-field effects in our controlled experiments.

The corresponding nonlinear regression models were developed to examine the models presented in cases 0–4. The models were constructed using a least squares optimization approach, where the dependent variable (log-transformed received energy) is expressed as a function of multiple predictor variables, including log-transformed propagation distance, squared frequency terms, and interaction terms reflecting frequency-dependent attenuation effects.

The regression parameters C_i_ were estimated using nonlinear least squares fitting via the Levenberg-Marquardt optimization algorithm, implemented in Python 3.11.4 scipy.optimize.curve_fit function. This iterative approach minimizes the sum of squared residuals between predicted and measured energy values, adapting step sizes dynamically to balance gradient descent and second-order approximations for convergence stability.

To improve model robustness, the predictor variables were normalized using z-score standardization before regression, ensuring that features with different magnitudes (e.g., logarithmic and quadratic terms) contribute proportionally to the optimization process. Additionally, multiple initial parameter guesses were tested to evaluate convergence behavior and ensure the stability of the fitted coefficients. The final model selection was based on the test set’s lowest mean squared error (MSE), confirming its predictive accuracy in estimating acoustic source strength across different frequencies and distances.

This section outlined the theoretical framework used to model acoustic source strength based on frequency-dependent attenuation principles. The nonlinear regression model leverages established acoustic transmission loss equations to estimate the initial intensity of a transmitted signal from recorded data. In the next section, we describe the experimental setup used to collect the dataset for training and validating these models.

## 3. Experimental Setup

To train a regression model for source strength (inverse attenuation), measuring and recording the energy of the acoustic signal at different distances from the source is required. The signal’s energy measured at the closest distance interval R0 is considered the source energy and hence will populate the results vector Y for the linear regression model. The energy measured at more considerable distance intervals R1, R2 (Ri), etc., will be the energy of signals attenuated along the travel distance Rt = (Ri − R0). Hence, these values and/or their logarithm, along with the distance (Ri − R0) and the frequency, will be used to construct the design matrix X.

Following this philosophy, the experimental setup consisted of two ultrasonic transducers (transmitter and receiver), a signal generator, a signal Oscilloscope, a mechanical spacer (a bar) that enables the transducers to be fixed at a fixed distance relative to each other, and a water tank. The position of the transducer in the water was such that the direct arrival at the receiver would be completely isolated in the time domain from any reflection from the boundaries. Also note that the distance between the transducers is more significant than 4.83 cm, corresponding to the far field threshold.

**Figure 1:**
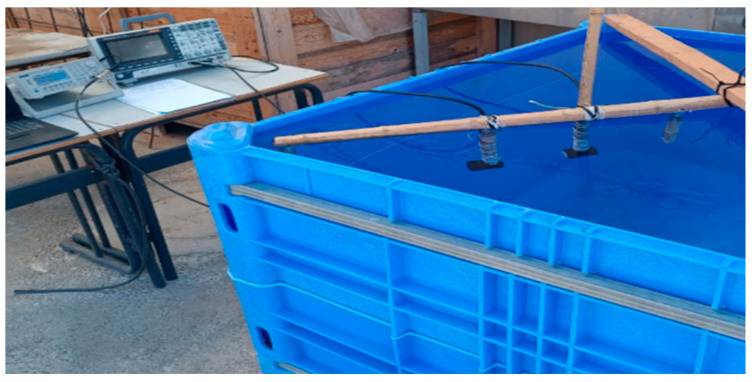
The experimental setup includes ultrasonic transducers (transmitter and receiver), a signal generator, a digital oscilloscope, a mechanical spacer (a bar), and a water tank.

**Figure 2:**
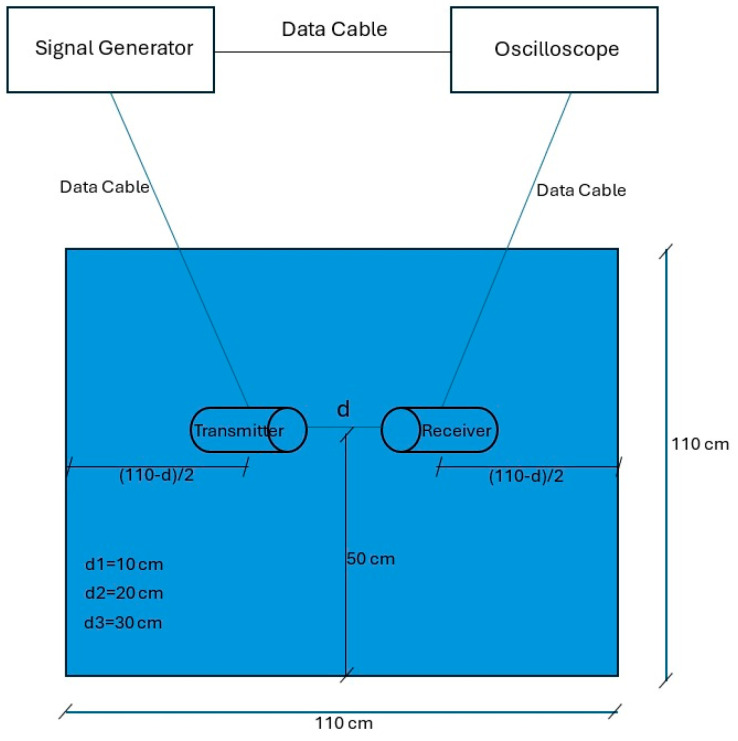
A schematic sketch for the experimental setup.

**Figure 3:**
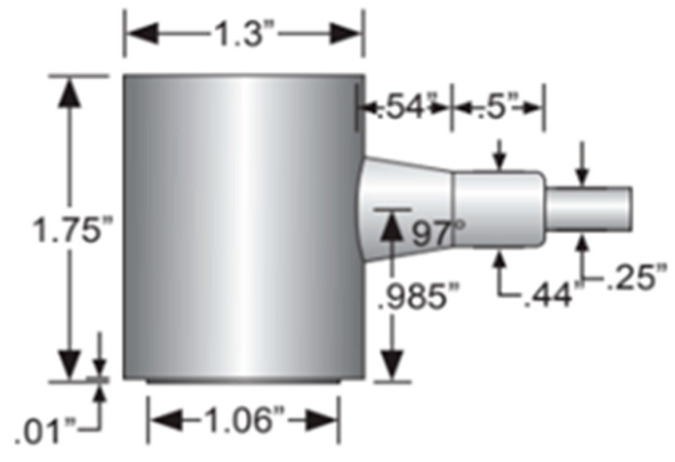
The dimensions of the transducers (MISTRAS Group, West Windsor, NJ, USA).

**Figure 4:**
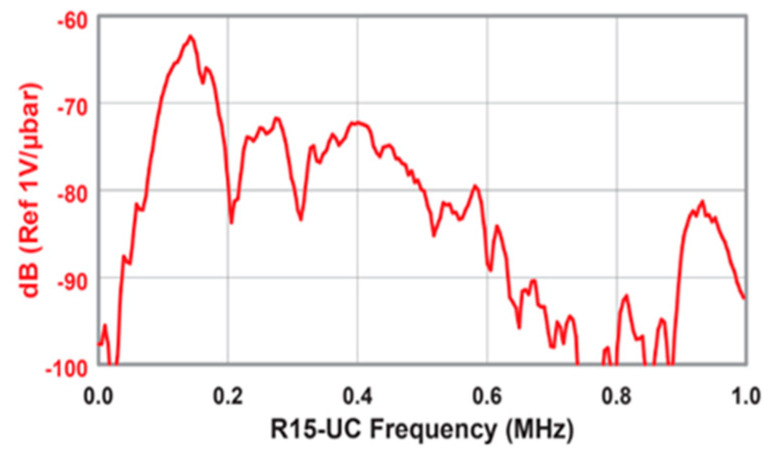
Transducer reception ranges in decibel values as a function of frequency. (MISTRAS Group, West Windsor, NJ, USA).

The transducers were placed facing each other for transmission and reception at 10 cm, 20 cm, and 30 cm. At each spacing, the signal generator was set to produce 3 and 5 cycles of monochromatic sinus with an amplitude of 10 volts. For each recording, the frequency was set manually. Separate files were recorded for each combination of cycles and frequencies, varying from 100–400 kHz and increasing in steps to 5 KHz. This resulted in 366 training examples (2 signal lengths, three distances, and 61 frequencies). Figure 1 consists of a photo of the experimental setup. Figure 2 includes a schematic drawing of the setup, and Figure 3 shows the transducer’s dimensions. For completeness, we also added the transducer reception curve in Figure 4:

The water used for the experiment was tap water from the Ashdod area with the following mineral composition as presented in Table 1 [29]:

### The Data Set

As described above, the data consisted of 366 recorded examples. The transmitted signal included 3 and 5 cycles of the monochromatic sinus with an amplitude of 10 volts and frequency varied from 100 KHz to 400 KHz in steps of 5 KHz. The signal was recorded at 10, 20, and 30 cm spacing between the transmitter and the receiver. Since the intensity at the transmission point cannot be measured, the recorded data was divided into 366 pairs, each with uniform frequency and cycles where the transmitted signal is considered the closer range record and the received signal is the far record (i.e., for a three-cycle 205 KHz pair recorded at 10 cm and 30 cm from the actual transmitter the 10 cm record will be the source and the 30cm record will be the receiver with 20 cm spacing. Similarly, pairs recorded at 20 and 30 cm or 10 and 20 cm will have a 10 cm spacing). The intensity of the signal was computed by integrating the square signal amplitude. Since we wished to find a gain (inverse attenuation) relation, the result vector y was comprised of the close-range intensity ($I_{0}$), and the far-range intensity ($I_{i}$) was a feature column in the design matrix X. The rest of the features included frequency and range, as detailed in the previous section describing the models.

The 366 examples were randomly shuffled and divided into a training set (75%) and a test set (25%).

## 4. Results

The results (trained coefficients and test set accuracy) of the nonlinear regression models, with the attenuation formulas presented in Section 2 for the different cases, starting from a preliminary coefficients value guess of zero for all coefficients, are summarized in Table 2.

**Figure 5:**
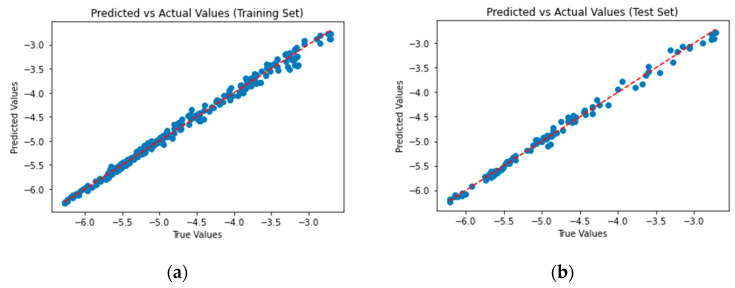
Predicted vs. Measured values for regression model case 4: (**a**) Training set, (**b**) Test set.

Case 4, which provided the best results, was also examined with different initial guesses for the coefficients. The comparison of trained coefficients for a preliminary guess of zero for all coefficients vs. a preliminary guess of 100 for all coefficients is provided in Table 3:

Note that a change in the preliminary guess did not affect accuracy. In addition, it did not affect the coefficients C1, C2, C7–C10. In particular, the same accuracy can be obtained when all the parameters C4–C6 and c4–c6 are zero, as seen in Table 3. When starting the regression with a zero guess for C4–C6 and c4–c6 and an arbitrary guess for all other coefficients, the model always converges with the following coefficients:

The predicted vs. actual log(I) values for the training set and test set for case 4 appear in Figure 5:

## 5. Discussion

The results showed that the regression model that provided the most accurate predictions over the test set was Case 4. A key observation from the analysis is that changes in the preliminary parameter guesses did not affect the model’s accuracy. Furthermore, the values of coefficients C1, C2, and C7–C10 remained consistent regardless of the initial guesses, whereas the initialization influenced the coefficients in the following term:

(13) $$\left[C_{3}f^{2}+C_{4}\frac{f^{2}c_{4}}{f^{2}+c_{4}^{2}}+C_{5}\frac{f^{2}c_{5}}{f^{2}+c_{5}^{2}}+C_{6}\frac{f^{2}c_{6}}{f^{2}+c_{6}^{2}}](R_{i}-1\right)$$

This means that for different preliminary guesses, C3–C6 and c4–c6 were adjusted so that this term is unchanged, and hence, all other coefficients were not affected. In particular, the same accuracy can be obtained when all the involved coefficients in expression 13 except C3 are zero as seen in Table 3 (and Table 4). When starting the regression with a zero guess for C4–C6 and c4–c6 and an arbitrary guess for all other coefficients, the model always converges to the same coefficients. Hence, case 4 can be simplified to our suggested formula Equation (14) for source strength for short distances in freshwater, which can replace the Thorpe relation. For fresh tap water in Israel, the relevant parameters appear as the trained parameters of Table 4.

(14) $$\log\left(I_{0}\right)=C_{1}\log\left(I_{i}\right)+C_{2}\log\left(R_{i}\right)+C_{3}f^{2}\left(R_{i}-1\right)+C_{7}+C_{8}I_{i}+C_{9}f+C_{10}f\left(R_{i}-1\right)$$

Note the features on the right side of the equation are normalized by the following equation:

(15) $$X^{\prime }=\frac{X-\mu }{\sigma }$$

where:

X is the original value of the feature for a specific training example.

μ is the mean of the feature values over all the training examples.

σ is the standard deviation of the feature over all the training examples.

X′ is a specific training example’s normalized (standardized) value.

The standard deviation and mean values of each feature appear in Table 5 and should be applied whenever using the trained model for prediction:

**Table 4:** Trained coefficients for case 4 with an arbitrary preliminary guess for all coefficients and zero preliminary guesses for C4–C6 and c4–c6.

Case 4 Preliminary Guess			
Preliminary Guess	Trained Coefficients	Preliminary Guess	Trained Coefficients
C1 = 7654	1.032682	C1 = 4356	1.032682
C2 = 15484	0.319061	C2 = −843611	0.319061
C3 = −685	0.186415	C3 = 5783	0.186415
C4 = 0	0	C4 = 0	0
C5 = 0	0	C5 = 0	0
C6 = 0	0	C6 = 0	0
c4 = 0	0	c4 = 0	0
c5 = 0	0	c5 = 0	0
c6 = 0	0	c6 = 0	0
C7 = −9857	−4.79983	C7 = 54789	−4.79983
C8 = 23890	−0.04989	C8 = 467	−0.04989
C9 = 120	0.103657	C9 = 49087	0.103657
C10 = 10	−0.23531	C10 = −596	−0.23531
MSE Test Set	0.0057574	MSE Test Set	0.0057574

**Table 5:** Mean value and standard deviation for feature normalization.

Features	Mean Value	Standard Deviation Value
$\log\left(I_{i}\right)$	−5.45121	0.87433
$\log\left(R_{i}\right)$	1.13261	0.131513
$f^{2}\left(R_{i}-1\right)$	940,386.9	707,901.4
$f^{2}$	71,307.3	45,476.67
$I_{i}$	$2.78\times 10^{-5}$	$8.31\times 10^{-5}$
f	251.5328	89.65783
$f\left(R_{i}-1\right)$	3325.547	1700.258

The developed model demonstrated a remarkable ability to predict source strength with high precision, independent of the initial parameter guesses. However, potential accuracy improvements, the possible incorporation of additional variables, and the model’s practical implications must be clarified. Moreover, the possible incorporation of the model into actual sonar and real-time monitoring systems must be emphasized. Finally, the limitations to implementing the model in dynamic and long-range scenarios must be clarified.

Refining feature selection to focus on the most relevant acoustic parameters can improve the regression model’s accuracy for estimating acoustic source intensity. Expanding the dataset with additional measurements across varying environmental conditions would enhance robustness and generalizability. Employing alternative regression methods, such as ensemble learning or nonlinear approaches, could better capture complex relationships in signal attenuation. Machine learning integration, particularly deep learning models, offers the potential for improving predictive accuracy by learning intricate dependencies in transmission loss, provided these models maintain physical interpretability.

Environmental factors such as temperature, salinity, and turbidity significantly impact sound propagation and could improve the model’s predictive capability. Temperature variations alter sound speed and refraction, which are essential for accurate long-range transmission loss estimates. Salinity influences sound absorption and impedance, particularly in estuarine and coastal waters where salinity gradients are pronounced. Turbidity contributes to scattering and attenuation, particularly at high frequencies, making it an essential factor in environments with suspended sediments or high biological activity. Including these variables would refine the model’s ability to account for real-world acoustic propagation effects, though the benefit depends on the availability of sufficiently diverse training data.

The model has significant implications for sonar operation, underwater navigation, and environmental monitoring. In sonar systems, it enhances calibration by providing a more accurate reference for source intensity, improving the interpretation of received echoes. This enables more precise target detection and classification, particularly in differentiating natural objects from artificial structures. In navigation, the model refines acoustic ranging techniques by improving transmission loss prediction under different environmental conditions, benefiting AUVs and long-baseline positioning systems. Environmental monitoring applications gain from the model’s ability to estimate and track noise sources, aiding in marine mammal detection, ship noise assessment, and regulatory compliance. Additionally, the model helps characterize the frequency-dependent acoustic properties of reflectors and targets independently of the attenuation effect. It enables improved classification of seabed materials, submerged objects, and biological targets based on their spectral responses.

Adapting the model to sonar and real-time monitoring systems would improve active and passive sonar performance by providing dynamic environmental corrections. Active sonar could use real-time environmental inputs to adjust transmission power and optimize detection range while minimizing unnecessary emissions. Passive sonar systems benefit from refined source localization, particularly in complex acoustic environments where variable transmission loss can obscure signal interpretation. Automated monitoring platforms, such as underwater acoustic observatories, could integrate the model to enhance real-time noise assessments and event detection by compensating for environmental variations in sound propagation. Adaptive sonar processing, incorporating model-driven adjustments to detection thresholds and filtering algorithms, would further improve the reliability and accuracy of acoustic detection systems.

Despite its strengths, the model faces limitations in dynamic and long-range scenarios where environmental fluctuations and complex propagation effects pose challenges. Rapid water temperature, salinity, and stratification changes can alter acoustic transmission characteristics, requiring frequent recalibration or real-time environmental updates. Long-range propagation introduces multi-path interference and absorption effects that a regression-based approach may not fully capture. Generalizability is another constraint, as the model’s accuracy depends on the representativeness of the training data, making deployment in unfamiliar environments less reliable without further validation. Computational efficiency is also a consideration for real-time applications, as complex models may require significant processing power that may not be available on embedded sonar systems. Addressing these limitations through hybrid modeling approaches that combine empirical regression with physics-based corrections could enhance adaptability and extend the model’s applicability to diverse underwater acoustic scenarios.

### 5.1. Scientific Novelty

This study presents a refined regression-based approach to inverse attenuation modeling in freshwater environments, addressing gaps in existing attenuation correction methods. While regression techniques have been widely used in acoustics, their targeted application to short-range freshwater conditions offers a methodology specifically designed to improve source strength estimation. Unlike traditional attenuation models that rely primarily on empirical or semi-empirical equations, this study demonstrates how data-driven regression can effectively account for frequency-dependent propagation effects and attenuation corrections in freshwater environments.

By systematically evaluating multiple regression models (Case 0 through Case 4), this study refines the understanding of geometric spreading, absorption, and frequency-dependent attenuation in near-field freshwater acoustics. The findings highlight the importance of incorporating additional correction terms to improve source strength estimation, particularly at shorter distances where classical models like Thorpe’s equation are less effective. This work contributes to underwater acoustics by providing an adaptable model that can be fine-tuned for different freshwater conditions, offering a more flexible alternative to rigid empirical formulations.

### 5.2. Future Research Directions

Further validation across diverse freshwater environments, brackish waters, and highly turbid conditions is essential to refine the model’s applicability. While the model effectively accounts for geometric spreading and absorption in controlled freshwater conditions, its performance in more complex environments—such as estuaries where salinity and suspended particles introduce additional attenuation mechanisms—should be examined. Extending the dataset to include different water compositions and environmental variables will improve its adaptability.

Integrating machine learning techniques represents a logical next step for enhancing model adaptability. A data-driven approach incorporating neural networks or ensemble learning could allow for real-time adjustments to regression parameters in response to dynamic environmental conditions, such as temperature fluctuations or varying sediment loads. While this study lays the foundation for regression-based attenuation modeling, hybridizing it with AI-driven adaptive models could improve predictive accuracy, particularly in real-time sonar and acoustic sensing applications.

## 6. Conclusions

This study presents a refined regression-based approach for estimating acoustic source strength in freshwater environments, specifically within the 100–400 kHz frequency range. The research addresses the inverse problem of attenuation correction, a critical challenge in underwater acoustics, by developing a nonlinear regression model that integrates key physical parameters such as signal energy, propagation path length, and frequency. The proposed methodology provides a simplified yet highly accurate alternative to conventional attenuation models, such as Thorpe’s equation, which is primarily suited for seawater conditions and long-distance acoustic propagation.

The model was validated through an experimental dataset comprising 366 controlled measurements using ultrasonic transducers in a freshwater tank under carefully regulated conditions. The results demonstrate that the proposed approach effectively predicts acoustic source strength while accounting for frequency-dependent attenuation, geometric spreading, and absorption losses. This capability makes the model particularly valuable for spectral analysis-based classification, sonar system calibration, underwater navigation, structural health monitoring of submerged infrastructure, and environmental monitoring applications.

A key scientific contribution of this study is its advancement in understanding the interplay between frequency-dependent attenuation and geometric spreading in freshwater systems. While most existing models are optimized for marine environments, the proposed model explicitly incorporates near-field effects, making it particularly well-suited for short-range acoustic propagation. The analysis across multiple regression cases (Cases 0–4) highlights the necessity of additional terms to accurately capture the short-range attenuation behavior in freshwater. The final optimized model (Case 4) provided the best predictive accuracy, leading to a generalized formula that can be used as a practical tool for attenuation correction in freshwater sonar applications.

Beyond theoretical advancements, the findings have broad implications for real-world applications. The 100–400 kHz frequency range is critical in various underwater acoustic technologies, as most MBES systems operate in this range. Applications include hydrographic surveys, sonar imaging, non-destructive testing of submerged structures, target identification, and aquatic environmental assessments. The study’s ability to enhance source strength estimation accuracy directly benefits these applications by reducing calibration errors and improving signal interpretation in complex analysis scenarios. The model’s flexibility suggests its potential scalability to aquatic environments beyond freshwater, including brackish and coastal systems where attenuation mechanisms differ from those in pure seawater.

While the results confirm the robustness and predictive accuracy of the proposed model, the study was conducted under controlled laboratory conditions, using fresh tap water with a known mineral composition. In real-world freshwater bodies, additional environmental factors—such as turbidity, suspended particles, and biological activity—can influence acoustic attenuation. Future research should, therefore, focus on validating the model in diverse freshwater environments, including lakes, reservoirs, and estuarine systems, to assess its adaptability to natural variability in water properties. Hence, another important future research direction involves extending the model to brackish and marine environments, where additional attenuation factors—such as salinity gradients, microbubble interactions, and temperature variations—play a more significant role. These factors could be incorporated into future model versions to expand their applicability across acoustic propagation conditions. Other frequency ranges beyond 400 kHz could be explored, particularly for advanced sonar applications requiring higher resolution or longer ranges.

One of the most significant areas for future enhancement is the integration of machine learning techniques to refine attenuation correction and source strength estimation further. By incorporating adaptive learning frameworks, the model could dynamically adjust its parameters based on real-time environmental changes, improving its applicability to operational sonar systems and autonomous underwater vehicles (AUVs). Machine learning-driven inverse problem solving has already demonstrated promising results in various fields, and its application to underwater acoustics could significantly enhance real-time signal processing and acoustic source localization.

In conclusion, this study presents an essential advancement in underwater acoustics, providing a practical, scientifically grounded, and highly accurate model for acoustic source strength estimation in freshwater environments and short-range applications. The research bridges theoretical innovation with real-world applications by refining traditional attenuation models and addressing the unique challenges of short-distance, high-frequency acoustic propagation. The results underscore the potential for further improvements through expanded datasets, environmental validation, and machine learning integration, ultimately contributing to the continued evolution of underwater acoustic modeling and its applications across scientific, industrial, and environmental domains.

## Figures and Tables

**Table 1:** Typical tap water composition in Ashdod.

Mineral/Ions	Typical Concentration (mg/L)	Health Ministry Standard
Sodium (Na^+^)	20–50	Up to 200
Chloride (Cl^−^)	20–100	Up to 250
Calcium (Ca^2^^+^)	20–40	Recommended 20–80
Magnesium (Mg^2^^+^)	5–20	Recommended 10–30
Bicarbonate (HCO_3_^−^)	20–100	No standard
Sulfates (SO_4_^2^^−^)	20–50	Up to 250
Nitrates (NO_3_^−^)	10–25	Up to 70
Potassium (K^+^)	1–5	No standard
TDS (Total Dissolved Solids)	100–250	Up to 1000

**Table 2:** Trained coefficients for case-to-case four regression models.

Trained Value					
Coefficient	Case 0	Case 1	Case 2	Case 3	Case 4
C1	0.918177	0.998122	0.971561	1.042503221	1.032682
C2	0.227291	0.204872	0.240315	0.232876191	0.319061
C3	0.023448	0.073103	0.002427	0.02497176	0.186415
C4	0	0	0	0	0
C5	0	0	0	0	0
C6	0	0	0	0	0
c4	0	0	0	0	0
c5	0	0	0	0	0
c6	0	0	0	0	0
C7	−4.81936	−4.79983	−4.79983	−4.79983167	−4.79983
C8	NA	−0.04481		−0.050928472	−0.04989
C9	NA	NA	0.058183	0.083561018	0.103657
C10	NA	NA	NA	NA	−0.23531
MSE Test Set	0.007413025142	0.007382779306	0.00781610234	0.006920767973	0.005757471523

**Table 3:** Trained coefficients for case4 with a preliminary guess of zero vs 100 for all the coefficients.

Coefficient Case 4	Zero Guess	100 Guess
C1	1.032682	1.032681577
C2	0.319061	0.31906107
C3	0.186415	−0.97954608
C4	0	4535.577075
C5	0	3324.608115
C6	0	2645.660003
c4	0	8895.980388
c5	0	8996.027717
c6	0	9232.742625
C7	−4.79983	−4.799831662
C8	−0.04989	−0.049891369
C9	0.103657	0.103656721
C10	−0.23531	−0.235306691
MSE Test Set	0.0057574	0.0057574

## Data Availability

The original contributions presented in the study are included in the article; further inquiries can be directed to the corresponding author.
